# 199. Fast Detection of Ceftazidime-avibactam-resistant *Enterobacterales* with VITEK-MS^TM^ Incorporating a Direct-on-target Microdroplet Growth Assay

**DOI:** 10.1093/ofid/ofad500.272

**Published:** 2023-11-27

**Authors:** Shenglei haung, Chengfeng qin, Bingchun Pu, Chunmei Zhou, Yan ma, Beili Wang, Baishen Pan, Bijie Hu, Wei Guo

**Affiliations:** Fudan university Zongshan hospital, shanghai, Shanghai, China; Institute of Microbial Epidemiology, Academy of Military Medical Sciences, Beijing, Beijing, China; Shanghai Rengji hospital, Shanghai, Shanghai, China; Fudan university Zhongshan hospital, Shanghai, Shanghai, China; Fudan university Zhongshan hospital, Shanghai, Shanghai, China; Fudan university Zhongshan hospital, Shanghai, Shanghai, China; Fudan university Zhongshan hospital, Shanghai, Shanghai, China; Department of Infectious Diseases, Zhongshan Hospital, Fudan University, Shanghai, Shanghai, China; Fudan university Zhongshan hospital, Shanghai, Shanghai, China

## Abstract

**Background:**

The direct-on-target microdroplet growth assay (DOT-MGA) was evaluated for its ability to rapidly detect ceftazidime-avibactam resistant *Enterobacterales*.

**Methods:**

In this study, 47 non-duplicate CRE isolates were used to determine unequivocally the performance of ceftazidime-avibatam resistance detection of 7 organisms found in clinical isolates. The isolates were incubated for 3 to 6 h in the presence and absence of 8 µg/mL ceftazidime-avibatam in the broth applied as microdroplets directly on to the matrix-assisted laser desorption ionization-time of flight mass spectrometry (MALDI-TOF MS) target spots. Following the incubation period, broth was separated from the microbes by pipette. MALDI-TOF MS was employed to evaluate the growth of isolates in each spot and to identify the microbes simultaneously. The micro-broth dilution method, recommended by EUCAST was used as a reference method. A PCR-based assay was carried out to evaluate the carbapenem resistance mechanisms by screening for the presence of various β-lactamase-encoding genes.

**Results:**

It was found that a 3 h incubation time was insufficient for MS to identify isolates. At this time-point, the identification of valid results of the growth control was only 25.0%. Therefore, the study of 3 h incubation times was not continued. For ceftazidime-avibactam-resistant *Enterobacter* strains, 83.7% of growth controls were successfully detected after the 4 h-time-point of incubation when the sensitivity and specificity of drug resistance detection were both 100%. Following incubation for 5 h, the rate of growth controls, sensitivity and specificity were 98.0%, 100% and 100%. When the incubation was extended to 6 h, the rate of growth controls, sensitivity and specificity reached an ideal 100%.

**Conclusion:**

The VITEK MS-based DOT-MGA is a fast, convenient and accurate in detecting *Enterobacterales* that is resistant to ceftazidime-avibatam within 5 to 6 h. The assay can also determine the resistance status of an isolate and the potential novel resistant mechanisms involved.

**Disclosures:**

**All Authors**: No reported disclosures

